# Editorial: Pre-interventional cardiac imaging

**DOI:** 10.3389/fcvm.2025.1605768

**Published:** 2025-04-23

**Authors:** Matthias Renker, Maxim Avanesov, Dominik Buckert

**Affiliations:** ^1^ Department of Cardiology, Campus Kerckhoff of the Justus-Liebig-University Giessen, Bad Nauheim, Germany; ^2^ German Centre for Cardiovascular Research (DZHK), Partner Site RheinMain, Frankfurt am Main, Germany; ^3^ Department of Diagnostic and Interventional Radiology and Nuclear Medicine, University Hospital Hamburg Eppendorf, Hamburg, Germany; ^4^ Department of Cardiology, Ulm University Heart Center, Ulm, Germany

**Keywords:** cardiac computed tomography (CCT), cardiac magnetic resonance (CMR), echocardiography, multimodality imaging, tissue characterization, preprocedural imaging, interventional guidance

**Editorial on the Research Topic**
Pre-interventional cardiac imaging

Cardiac imaging techniques are essential for ensuring optimal results and increasing patient safety in the context of invasive cardiac procedures. The field of cardiac imaging has undergone significant advancements primarily through enhanced diagnostic precision that allows for sophisticated peri-procedural evaluation. Among the most relevant imaging modalities, echocardiography, cardiac magnetic resonance imaging (CMR) and cardiac computed tomography (CCT) have revolutionized risk assessment, procedural guidance, and post-interventional assessment and are therefore emphasized here.

Echocardiography is the cornerstone of cardiac imaging as it is an easily accessible and portable modality for functional assessment offering dynamic visualization of cardiac motion, valve function, and also hemodynamics. While transthoracic echocardiography allows for immediate bedside evaluations in routine clinical practice, transesophageal echocardiography (TEE) also facilitates guidance of interventional procedures by way of further enhanced diagnostic accuracy and more particularly featuring high-resolution images of posterior cardiac structures (1).

CMR provides advanced tissue characterization with its ability to quantitatively assess focal and diffuse interstitial myocardial fibrosis, edema, perfusion, extracellular volume, and viability in a variety of different diseases (2–6). The ability to quantify cardiac ventricular volumes and function as well as characterize myocardial tissue composition by novel mapping techniques (7) and 4D flow dynamics (8) with high reproducibility, makes CMR the reference standard for comprehensive cardiac assessment without ionizing radiation.

CCT provides anatomical visualization with high spatial resolution, allowing for detailed assessment of coronary artery disease and cardiac anomalies (9). Based on its central role in the evaluation of patients prior to transcatheter aortic valve implantation and other structural heart interventions, CCT is nowadays essential for pre-procedural structural heart disease management (10, 11, 12).

Within this research collection, we present and discuss high-quality contributions from the field of cardiovascular imaging with focus on its pre-interventional role and beyond, aiming to cover recent advancements in the field, to familiarize readers with the current state-of-the-art and to provide an outlook into the future. The featured articles include original research articles, reviews as well as case reports highlighting the wide area of application covered by cardiac imaging.

In the first of three selected case reports, Baltodano-Arellano et al. present a rare instance of a cardiac hematic cyst, detailing its imaging characteristics, differential diagnoses and surgical treatment. The accompanying literature review contextualizes the case within the broader spectrum of cardiac masses, offering valuable insights for clinicians encountering similar presentations. In their case report, Tian et al. describe an innovative approach to endoluminal removal of a superior vena cava filter, which was based on pre-interventional CT imaging. The findings provide practical insights for assessing and managing superior vena cava filter complications. In yet another case report, Kang et al. present a rare instance of mechanical hemolysis due to left ventricular (LV) outflow tract obstruction following aortic valve replacement. The successful resolution through transapical beating-heart septal myectomy underscores the role of advanced cardiac imaging in identifying and addressing procedural complications.

The original research study by Pan et al. examines the anatomical details of the mitral isthmus and its proximity to the esophagus, offering insights relevant to atrial fibrillation ablation procedures. Based on CCT, the authors provide a detailed assessment of spatial variations, which can inform procedural strategies to enhance safety and efficacy in catheter-based interventions.

Zheng et al. address the risk and prognostic significance of LV ejection fraction decline in asymptomatic patients with primary mitral regurgitation. Based on risk stratification with cardiac imaging, the study stresses the necessity of timely intervention before irreversible ventricular dysfunction develops.

The role of TEE in percutaneous closure of patent foramen ovale is the focus of a study by Luo et al. It introduces a novel method aimed at enhancing diagnostic accuracy and procedural safety, which has implications for reducing complications and optimizing outcomes in structural heart disease interventions.

In another interesting study, Zamani-Aliabadi et al. investigate the utility of 4D flow CMR in quantifying left-to-right shunting in pediatric ventricular septal defect. Comparing its accuracy with right heart catheterization, their research emphasized the potential of noninvasive cardiac imaging in congenital heart disease assessment and management including interventional closure.

The original research by Kong et al. evaluates the diagnostic performance of stress dynamic CT myocardial perfusion imaging in detecting myocardial ischemia. The study highlights the comparative efficacy of absolute and relative myocardial blood flow parameters in identifying hemodynamically significant coronary artery disease.

In the original research by Liu et al., investigators explore the implications of self-reported SARS-CoV-2 antibody positivity on cardiac morphology and function using CMR imaging. Their findings contribute to the growing body of knowledge on post-viral cardiac involvement, emphasizing the role of imaging surveillance in affected populations and their risk of LV hypertrophy potentially requiring medical intervention.

Finally, the elegant review by Stoiculescu et al. synthesizes current evidence on advanced imaging techniques in optimizing cardiac resynchronization therapy. It discusses the role of echocardiography, CMR and also nuclear imaging in patient selection, lead placement, and response prediction, highlighting how multimodality cardiac imaging refines therapeutic outcomes in interventional heart failure treatment.

All of the articles featured in this research collection exemplify the pivotal role of pre-interventional cardiac imaging for patient safety, procedural success and therapeutic outcomes. Moreover, imaging-based risk stratification, guidance of complex interventions and refined therapeutic decision-making are highlighted by each one of the contributions demonstrating the ongoing evolution of cardiovascular imaging in contemporary practice. As technological advancements continue to reshape the landscape of cardiac imaging, further adoption in clinical practice, standardization of image interpretation among cardiac imaging specialists and ongoing research focused on these advanced diagnostic modalities will be vital to ensure optimized patient care.
